# Interplay Between Oral Microbiota and Mouth Health in People Living With HIV Under Antiretroviral Therapy With or Without Periodontitis

**DOI:** 10.1155/ijod/8794149

**Published:** 2026-04-09

**Authors:** Diana Estefania Ramos Peña, Oulfa Boussetta-Charfi, Angéline Antezack, Nacera Amroune, Philippe Colson, Virginie Monnet-Corti, Rafael Simone Saia, Johann Guillemot, Bruno Pozzetto, Sylvie Pillet, Bernard La Scola, Thomas Bourlet, Ana Carolina Fragoso Motta

**Affiliations:** ^1^ Department of Stomatology, School of Dentistry, University of São Paulo, São Paulo, SP, Brazil, usp.br; ^2^ Team Mucosal Immunity and Pathogen Agents (GIMAP), International Centre of Research in Infectiology (CIRI), INSERM U1111, Ecole Nationale Supérieure de Lyon, University of Lyon, University of Saint-Etienne, Saint-Etienne, France, universite-lyon.fr; ^3^ Department of Infectious Agents and Hygiene, University-Hospital of Saint-Etienne, Saint-Etienne, France, chu-st-etienne.fr; ^4^ Microbes, Evolution, Phylogeny and Infection (MEPHI), IHU-Méditerranée Infection, Marseille, France, mediterranee-infection.com; ^5^ Dental School, Faculty of Medical and Paramedical Sciences, Aix-Marseille Univ, Marseille, France, univ-amu.fr; ^6^ PROMOD-Odontology Department, Timone Hospital, Marseille, France, ap-hm.fr; ^7^ UR MEPHI (Research Unit D-258, Microbes Evolution Phylogeny and Infection), Aix Marseille University/Laboratory of Infectious Agents, Timone Hospital, Assistance Publique-Hôpitaux de Marseille/IHU (Institut Hospitalo Universitaire) -Méditerranée Infection, Marseille, France, ap-hm.fr; ^8^ Laboratory of Intestinal Pathophysiology, Department of Physiology, Ribeirão Preto Medical School, University of São Paulo, Ribeirão Preto, SP, Brazil, usp.br; ^9^ Department of Stomatology, Public Health and Forensic Dentistry, School of Dentistry of Ribeirão Preto, University of São Paulo, Ribeirão Preto, SP, Brazil, usp.br

**Keywords:** dysbiosis, HIV-1 infection, inflammatory markers, metagenomic sequence analysis, oral microbiota, periodontal diseases/therapy, periodontitis, saliva

## Abstract

People living with HIV (PLWH) in combined antiretroviral therapy (cART) face microbiota shifts linked to immune status, ART regimen, and periodontal diseases, which are capable of inducing local and systemic inflammation. This study aimed to analyze the oral microbial community composition in PLWH under cART with (*n* = 24) or without (*n* = 25) periodontitis using shotgun metagenomic sequence analysis, and describe the interaction between bacterial species, clinical and immunological parameters, and the response to nonsurgical periodontal therapy (NSPT). Saliva samples were collected at baseline for both groups, and 30 days after NSPT for the periodontitis group. Within the periodontitis group, all periodontal parameters presented highly significant improvement after NSPT when compared to baseline. The gingival microbiota did not differ significantly between patients with periodontitis and controls; however, a wider range of bacterial species was found in the microbiota of the periodontitis group compared to the control group, while post‐treatment the periodontitis group presented an alpha diversity intermediate between the two former groups. Regarding the distribution of the different bacterial species, *Porphyromonas gingivalis* was found significantly enriched in the periodontitis group, along with different *Treponema* sp., *Fretibacterium fastidiosum*, *Campylobacter rectus*, *Bacteroides zoogleoformans*, *Tannerella forsythia*, and *Porphyromonas endodontalis*. Correlations between seven inflammatory markers and seven periodontitis‐related bacterial taxa were found for saliva in the group of periodontitis patients, which was not the case in controls; interestingly, the profiles after NSPT showed intermediate results. By contrast to saliva, the inflammatory markers of periodontitis patients showed no marked differences in blood plasma, except for TNF‐alpha and partly IL‐4. In view of the fact that oral microbial imbalance may contribute not only to local disease but also to systemic immune activation in the course of HIV‐1 infection, reinforcing the importance of maintaining periodontal health represents a part not to be neglected for an optimal management of PLWH.

## 1. Introduction

Oral microbiota and the host interact through structured biofilm, microbial metabolites, and virulence factors that trigger the innate and adaptative immune response. Mucosa homeostasis maintains the balance within this interaction, while an altered dysbiotic microbiota is able to produce local tissue destruction and activation of systemic inflammation [1–4]. The oral cavity provides an optimal environment for the development of structured polymicrobial biofilm that has the capacity to determine health or diseased outcomes, and that can be heavily influenced by the systemic status of the patient [3, 4].

Despite common delivery of combined antiretroviral therapy (cART), people living with HIV (PLWH) still experience several oral conditions, ranging from candidiasis to periodontal diseases and neoplasms, which may reflect systemic immune defect and can affect their quality of life [5, 6]. In view of this, the oral microbiota has emerged as a marker and a potential mediator of local and systemic diseases. Indeed, multivariate studies suggest that HIV/ART produces measurable, but generally modest, effects on oral microbial community structure when compared to local alterations such as those induced by periodontal diseases, smoking, and dental caries [7, 8]. These observations motivate a site‐specific investigation at salivary and subgingival ecosystems in the ART era.

Saliva integrates microbial characteristics from multiple oral niches, and cohort studies have indicated that HIV infection is associated with shifts in salivary bacterial diversity and composition that only partially normalize after ART initiation [9–13]. ART regimen may also interfere in the oral microbiota. In a longitudinal clinical trial, initiation of tenofovir/emtricitabine/efavirenz was followed by complex salivary microbiome changes whose magnitude and direction varied with immune reconstitution, underscoring the influence of CD4 cell count recovery on oral microbial communities [11]. Cross‐sectional and longitudinal studies further reported that specific taxa, such as *Neisseria*, *Haemophilus*, and *Prevotella*, correlated with CD4 cell count and viremia, and that some differences persisted despite viral suppression [10, 12]. Beyond bacteria, the salivary mycobiome is also altered in HIV‐1 infection and appears to track virologic and immunologic status, hinting at broader ecological disruption [14].

While the oral microbiota is generally considered less influenced by HIV‐1 infection than the gut microbiota [15, 16], recurrent and significant changes in its composition have been identified in PLWH, notably in the presence of periodontitis [9, 17, 18]. On a systemic level, chronic oral inflammation generates pro‐inflammatory cytokines, including IL‐6 and TNF‐α, and enhanced activity of IL‐17, which, in combination with dysbiotic inflammation, can enhance the development or the magnification of systemic conditions [19]. Collectively, these reported results relative to salivary microbiota point to HIV/ART‐linked dysbiosis modulated by the immune response and the local inflammatory process. Using shotgun DNA/RNA metagenomic sequencing of total saliva, the aims of this study were (1) to compare the oral microbial community composition in PLWH under cART with and without periodontitis (beta diversity and taxa abundances at baseline), (2) to evaluate microbiome changes 30 days after nonsurgical periodontal therapy (NSPT) in the periodontitis group, and (3) to examine associations between periodontitis‐related bacterial taxa, clinical periodontal indices, and inflammatory markers in total saliva and blood plasma.

## 2. Materials and Methods

### 2.1. Patients Selection and Sample Size Calculation

Fifty‐four PLWH under cART, were included in this study at the School of Dentistry of Ribeirao Preto, Brazil, between July 2019 and March 2023 and classified according to the periodontal status in periodontitis group (*n* = 26) and control group without periodontitis (*n* = 28). All patients underwent a full‐mouth periodontal examination: some subjects of the control group exhibited localized gingivitis but none of them showed signs of periodontitis; patients of the periodontitis group received a NSPT by a certified dentist, as previously described [18].

The inclusion criteria for both groups were as follows: (i) concerning the virological criteria, HIV‐1‐infected patients under active cART, with CD4 cell count >200 cells/mm^3^ and undetectable HIV RNA load at the threshold of 40 copies/mL for at least 6 months before the recruitment date, and (ii) concerning the dental criteria, presence of at least 15 natural teeth for subjects of both groups, confirmed periodontitis diagnosis based on at least two sites with periodontal probing pocket depth (PPD) of ≥5 mm and clinical attachment level (CAL) of ≥5 mm with positive bleeding on probing (BOP) for patients of the periodontitis group, and confirmed absence of periodontitis based on PPD and CAL levels of ≤4 mm for subjects of the control group.

The exclusion criteria for both groups were history of periodontal therapy or antibiotic treatment in the previous 6 months, systemic diseases that could affect the progression of periodontitis (e.g., diabetes mellitus, hypertension, and cardiovascular diseases), extensive prosthetic restorations, and long‐term administration of anti‐inflammatory drugs.

The number of participants was established using the G^∗^ Power software version 3.0.10 (Christian‐Albrechts University, Kiel, Germany). Assuming an effect size of 80%, a significance level of 5% and test power of 80%, the estimated sample size was 50 HIV‐1 infected individuals, 25 with periodontitis and 25 without periodontitis.

For the purpose of this study related to saliva microbiota, five patients who refused to provide this sample were excluded secondarily, three in the control group and two in the periodontitis group, which resulted in a remaining effective of 49 participants. As this missingness was unrelated to the study outcomes and did not affect the balance between groups, we considered that no significant bias was introduced in the study results.

### 2.2. Ethics Statement

This study was approved by the Brazilian National Committee of Ethics in Research (CONEP) on the first of March 2018 (CAAE: 81355517.1.0000.5419). All patients provided written informed consent.

### 2.3. Sample Collection

Biological samples of blood plasma, peripheral blood mononuclear cells (PBMCs), and total saliva were collected from each patient as previously described [20]. For the control group the samples were collected only once whereas, for the periodontitis group, the samples were collected at baseline (T0, which corresponds to the date of treatment), and 30 days after the institution of NSPT (T1). The latter period of follow‐up was selected to ensure clinical periodontal stabilization after NSPT, while securing early microbiological changes. All samples were stored at −80°C until processing.

### 2.4. Cytometric Bead Array (CBA)

A panel of cytokine concentration, including IL‐2, IL‐4, IL‐6, IL‐10, IL‐17A, TNF‐α, and IFN‐γ in saliva and plasma, was determined by using the Human Th1/Th2/Th17 CBA kit (BD Biosciences, San Jose, CA, USA) to detect the expression of cytokines according to the manufacturer’s instructions. All samples were acquired on a Cytek Aurora spectral flow cytometer (Cytek Biosciences, Fremont, CA, USA) and analyzed using FlowJo software version 10 (BD Biosciences).

### 2.5. HIV DNA Load

HIV DNA load was measured in PBMCs with the Biocentric Generic HIV DNA Cell kit by real time PCR (Bruker/Hain Lifescience, Nehren, Germany). Results were normalized and expressed in copies per 10^6^ cells. For PBMCs, frozen samples were thawed and 200 μL of each sample were thinned with an equal volume of Digest‐EUR (Eurobio Scientific, Les Ulis, France) to reduce viscosity, then vortexed and incubated for 10 min. The samples were then centrifuged at high speed for 5 min at 1000 g to reduce cellular contamination. Before DNA extraction, an enzymatic digestion step was performed by adding 10 μL of DNase to each sample for 20 min at room temperature for removing free human DNA. Afterwards, each sample was treated with 50 μL of proteinase K for 10 min at 50°C. Finally, DNA extraction was carried out using the EasyMag device (bioMérieux, Marcy‐l’Etoile, France) following the manufacturer’s instructions.

### 2.6. Microbiota Analysis in Saliva

DNA extraction from saliva samples was performed using the EasyMag device (bioMérieux) following the manufacturer’s instructions. The frozen DNA extracts were then processed for next‐generation sequencing using the Illumina Novaseq 6000 system (Illumina Inc., San Diego, CA, USA). Briefly, DNA samples were quantified with Qubit 4 fluorometer (Invitrogen, Waltham, MA, USA) and the sequencing libraries were prepared using the Nextera XT DNA protocol (Illumina). After amplification by PCR, the products were purified using Clean NGS magnetic beads kit (CleanNA, Waddinxveen, The Netherlands) to remove very short DNA fragments and retain those of adequate size for sequencing. Library quality was checked with the Agilent High Sensitivity DNA Kit (Bioanalyzer Agilent Technologies, Santa Clara, CA, USA) to determine DNA size, while DNA concentration was measured again with the Qubit 4 fluorometer. Finally, dilutions were performed to ensure homogeneous concentration of all samples. Pool preparation at 1 nM, loading through an S1 flow cell on the NovaSeq 6000 sequencer (Illumina Inc.) was performed with a reading of 2 × 150 base pairs (bp) following the manufacturer’s instructions.

### 2.7. Statistical Analysis and Sequence Data Processing

Demographic and clinical data were evaluated using descriptive statistics. Categorical variables were expressed by frequencies and percentages, and the continuous variables were expressed by means and standard deviation, or median and interquartile ranges, as appropriated. Data were checked for normality using the Shapiro–Wilk test. Fisher exact test was used to compare categorical variables. The parametric *t*‐test or nonparametric Mann–Whitney test were used to compare continuous outcomes between the periodontitis and control groups. The Wilcoxon test was used to compare non‐normally distributed continuous variables before and after NSPT in the periodontitis group. Descriptive and statistical analyses were performed using Jamovi software version 2.6.19.

For the metagenomic workflow, raw paired‐end sequencing reads were processed on the Galaxy Europe platform (https://usegalaxy.eu/). Quality assessment was performed with FastQC, and reads were trimmed with Trimmomatic (https://github.com/timflutre/trimmomatic) to remove adapters and low‐quality bases. Taxonomic classification was conducted using Kraken2 (https://github.com/DerrickWood/kraken2) with the RefSeq Standard‐Full database (including archaea, bacteria, viruses, plasmids, human, and UniVec_ Core; version 2022‐06‐07). Classification outputs were summarized with Kraken‐report and explored interactively using Pavian (https://github.com/fbreitwieser/pavian). Downstream analyses were performed in R (version 4.4.1, 2024‐06‐14) using the phyloseq package for data handling and diversity analysis, and DESeq2 for differential abundance testing, where results were expressed as log_2_ fold changes with adjusted *p*‐values. A Permanova analysis was used for evaluation of the beta‐diversity using Adonis function from the Vegan package (version 2.7‐1). The correlations between bacterial species and clinical or immunological parameters were evaluated using Spearman correlation test with the Benjamini–Hochbergalse false discovery rate (FDR) procedure for correcting multiple testing, and illustrated graphically with heatmap matrices.

## 3. Results

### 3.1. Patients

From the 54 PLWH initially included in this study, five (three in the control group and two in the periodontitis group) were excluded due to missing biological samples. From the 73 collected saliva samples in the remaining 49 participants, 68 samples could be analyzed reliably after completion of genomic DNA extraction and sequencing (25 in the control group, 24 in the periodontitis group before treatment, and 19 in the periodontitis group after treatment). The characteristics of the subjects included in the final analysis are presented in Table 1 (clinical profile and immunological parameters) and Table 2 (periodontal parameters). There was no significant difference regarding sociodemographic characteristics, HIV, or cART history between control and periodontitis subjects (Table 1). Within the periodontitis group, all periodontal parameters presented highly significant improvement after NSPT (T1) when compared to T0 (Table 2). It is particularly important to notice an 80% decrease of the mean of persistent PPD >5 mm sites 30 days after NSPT introduction.

**Table 1:** Clinical profile and immunological parameters of people living with HIV with (periodontitis) and without (control) periodontitis analyzed in this study.

Variable	Controls (*N* = 25)	Periodontitis (*N* = 24)	*p*‐Value
Age in years (mean ± SD)	43.9 ± 10.9	46.3 ± 9.5	NS^a^
Weight in kg (mean ± SD)	83.5 ± 24.4	79.9 ± 17.1	NS^b^
Smoking			
No	15 (60)	14 (58)	NS^c^
Former	7 (28)	6 (25)	
Yes	3 (12)	4 (17)	
Alcoholism			
No	10 (40)	3 (13)	NS^c^
Former	4 (16)	6 (25)	
Socially	7 (28)	11 (46)	
Yes	4 (16)	4 (16)	
Drug use			
No	21 (84)	18 (75)	NS^c^
Former	1 (4)	5 (21)	
Yes	3 (12)	1 (4)	
History of other STI			
No	18 (72)	12 (50)	NS^c^
Yes	7 (28)	12 (50)	
HIV‐1 diagnosed time in months (mean ± SD)	519.5 ± 572.6	393.87 ± 475.02	NS^b^
Time without cART until treatment initiation (mean ± SD)	70.8 ± 252.7	16.5 ± 28.3	NS^b^
Time under cART in months (mean ± SD)	448.7 ± 555.4	377.4 ± 480.9	NS^b^
Time with undetectable viral load in months (mean ± SD)	66.0 ± 49.7	81.5 ± 54.1	NS^b^
CDC classification			
1	20 (80)	20 (83)	NS^c^
2	5 (20)	4 (17)	
3	0	0	
CD4 nadir (mean ± SD)	154.3 ± 186.0	266.8 ± 233.8	NS^b^

*Note:* Data presented as number of subjects (%), unless otherwise indicated.

Abbreviations: cART, combined antiretroviral therapy; CDC, Centers for Disease Control and Prevention; NS, not significant; SD, standard deviation; STI, sexually‐transmitted infection.

^a^ *t* test.

^b^Mann–Whitney test.

^c^Fisher’s exact test.

**Table 2:** HIV DNA load and periodontal parameters of people living with HIV with (periodontitis) and without (control) periodontitis analyzed in this study.

Variable	Controls (*N* = 25)	Periodontitis before treatment (*N* = 24)	Periodontitis after treatment (*N* = 19)	*p*‐Value^a^
Proviral HIV DNA in PBMC in copies/10^6^ cells (mean ± SD)	19,246.329 ± 16,839.871	79,232.235 ± 301,236.428	27,048.94 ± 41,780.55	NS^b^
				NS^c^
Number of teeth (mean ± SD)	26.0 ± 4.2	25.3 ± 4.67	24.5 ± 4.3	NS^b^
				**0.034** ^c^
Periodontal probing depth (PPD) in mm, median (interquartile)	2.02 (1.83–2.17)	2.72 (2.49–3.14)	2.2 (1.9–2.2)	**<0.001** ^b^
				**<0.001** ^c^
PPD > 5 mm (mean sites ± SD)	—	18.0 ± 19.9	3.6 ± 2.6	**<0.001** ^c^
Clinical attachment level (CAL) in mm (mean ± SD)	2.33 ± 0.5	3.42 ± 1.2	2.8 ± 0.68	**<0.001** ^b^
				**<0.001** ^c^
Bleeding on probing (BOP) in % (mean ± SD)	21.4 ± 17.7	43.8 ± 22.7	12.5 ± 8.6	**0.001** ^b^
				**<0.001** ^c^
Plaque index (PI) in % (mean ± SD)	52.2 ± 25.7	69.4 ± 22.9	28.7 ± 17.6	**0.013** ^b^
				**<0.001** ^c^

*Note*: Bold value means a significant difference between the two groups at the 0.05 level.

Abbreviations: NS, not significant; SD, standard deviation.

^a^When data are available for the three groups, the first line corresponds to the comparison between controls (*N* = 25) and patients with periodontitis at baseline (*N* = 24) and the second line to the comparison of 19 periodontitis patients at baseline and after nonsurgical periodontal treatment.

^b^Mann–Whitney test.

^c^Wilcoxon test.

### 3.2. Microbial Diversity in Periodontitis and Control PLWH

In the analysis of the whole sample set, including those from both groups and time points, Figure 1 illustrates beta‐diversity and relative abundance. Results using a Permanova analysis show that the beta‐diversity of gingival microbiota did not differ significantly between patients with periodontitis and controls (*p* = 0.306 by Adonis test) since all bacteria identified in the control group were present in the periodontitis group in a similar proportion (Figure 1A). Both groups of patients exhibited a high proportion of *Actinomyces* sp., *Rothia* sp., and *Selenomonas* sp. (Figure 1B). In the periodontitis group after NSPT, *Rothia* sp., *Staphylococcus aureus*, and *Lautropia mirabilis* were also abundant; this group presented an enrichment in *Neisseria* sp. reaching a level similar to that of the control group (Figure 1B). As shown in Figure 1C, *Porphyromonas gingivalis* was found significantly enriched in the periodontitis group, along with different *Treponema* species, *Fretibacterium fastidiosum*, *Campylobacter rectus*, *Bacteroides zoogleoformans*, *Tannerella forsythia*, and *Porphyromonas endodontalis*, while the control group presented enrichment of *Rothia mucilaginosa*.

Figure 1(A) Beta‐diversity based principal component analysis (PCA) plot of saliva microbiota of periodontitis before and after nonsurgical periodontal therapy (NSPT) and control groups. (B) Relative abundance of the main bacterial species for all samples of the periodontitis before and after NSPT and control groups. Numbers in abscisses correspond to the number affected to each participant of the study. The asterisk following a number means that the corresponding patient was tested again after NSPT (a few data are missing after treatment). (C) Volcano plot of the main bacterial species of periodontitis and control groups of log_2_ fold‐change > 1.

**Figure:**
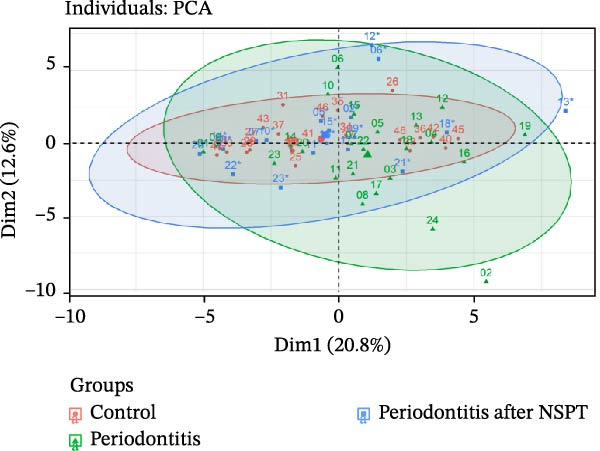
(A)

**Figure:**
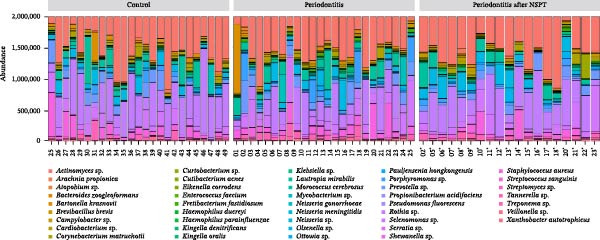
(B)

**Figure:**
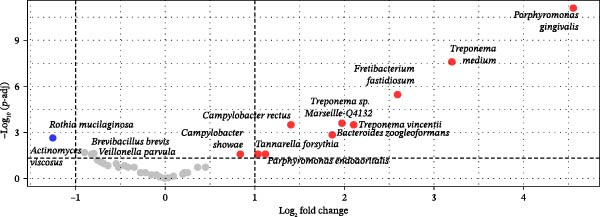
(C)

**Figure 2:**
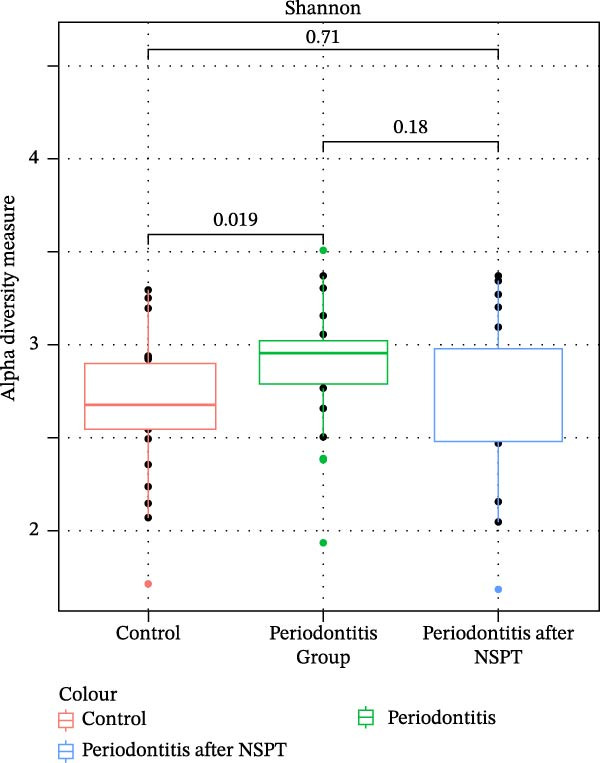
Shannon index alpha‐diversity of saliva microbiota in periodontitis before and after nonsurgical periodontal therapy (NSPT) and control groups.

As for alpha diversity (Figure 2), the periodontitis group had a wider range of bacterial species compared to the control group as calculated by the Shannon index (*p* = 0.019). The periodontitis group after treatment presented a decrease in alpha diversity that became more similar to the one of the control group (not significant *p*‐value). Alpha diversity in relation to lifestyle habits, such as smoking and alcohol consumption, and use of drugs did not show significant difference. Also, no difference regarding history of other sexually‐transmitted diseases was found. Regarding the use of cART, no significant association was found with alpha diversity for both groups. Although not statistically significant, a lower diversity was observed in patients under protease inhibitors and integrase inhibitors, while an increased diversity was observed in patients under nucleos(t)ide reverse transcriptase inhibitors and non‐nucleoside analogs.

### 3.3. Correlation Analysis Between Some Bacterial Taxa and Immunological Markers

**Figure 3:**
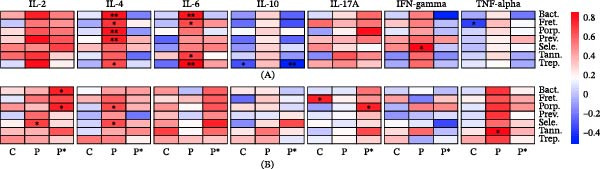
Heatmap of Spearman correlations between the relative abundance of seven bacterial taxa and the level of seven cytokines (IL‐2, IL‐4, IL‐6, IL‐10, IL‐17A, IFN‐γ, and TNF‐α) in whole saliva (A) and plasma (B). The three groups of subjects are designed by C for controls, P for periodontitis patients at baseline, and P^∗^ for periodontitis patients 30 days after nonsurgical periodontal therapy. The correlations were established according to the whole oral microbiota described in Figure 1B but only seven taxa whose abundance is known to be frequently associated with periodontitis are shown on the panels: Bact. = *Bacteroides zoogloformans*, Fret. = *Fretibacterium fastidiosum*, Porp. = *Porphyromonas* sp., Prev. = *Prevotella* sp., Sele. = *Selenomonas* sp., Tann. = *Tannerella* sp., and Trep. = *Treponema* sp. Statistically significant correlations are indicated by asterisks:  ^∗^ *p* < 0.05;  ^∗∗^ *p* < 0.001;  ^∗∗∗^ *p* < 0.0001.

In this section, we grouped some oral bacteria in genera and we established heatmaps of Spearman correlations between the bacterial taxa and some immunological markers of interest. This analysis was performed for the different inflammatory markers measured in saliva and blood plasma. Even if the correlations were performed on the whole oral microbiota (Figure 1B), we selected in Figure 3 seven bacterial taxa that were shown in previous reviews based on meta‐analyses [21–23] to be linked to periodontitis, namely *Bacteroides zoogleoformans*, *Fretibacterium fastidiosum*, *Porphyromonas* sp., *Prevotella* sp., *Selenomonas* sp., *Tannerella* sp., and *Treponema* sp. Interestingly, these seven taxa were shown to be globally correlated to the seven inflammatory markers measured in the saliva of periodontitis patients by comparison to controls. In addition, after treatment, most profiles tended to become closer to that of controls, except for IL‐2, IL‐17A, and partly IL‐6 (Figure 3A). The overall correlation observed in saliva between the seven taxa and the inflammatory markers was not confirmed in blood plasma of periodontitis patients, except for TNF‐alpha and partly for IL‐4 that exhibited marked difference with regard to controls (Figure 3B).

## 4. Discussion

By contrast to ancient microbiota studies performed in HIV‐1 patients that targeted the immunological weaknesses of the host, the present study focused on PLWH with or without periodontitis under active and effective cART without stigmata of systemic immunosuppression, which is more in line with the current controlled state of the HIV‐1 pandemic [11, 24]. In addition, little is known about the microbial changes observed after NSPT in PLWH with periodontitis. In the HIV‐1 context, shifts in the oral microbiota and mucosal immunity have been described, including a tendency to dysbiosis [12, 25], periodontal disease [17, 18, 26], and opportunistic infections [4] that could increase the systemic inflammation. HIV‐1 infection and its immunological state can modify oral microbial communities, commonly presenting altered bacterial diversity with enrichment of pathogenic species [7, 13, 18]. Interestingly, our results demonstrate the presence of similar bacterial communities in PLWH whatever the presence or absence of periodontitis. However, a higher Shannon index and an increased abundance of gingivitis‐ and periodontitis‐related bacterial genera (*P. gingivalis*, *T. medium*, and *F. fastidiosum*) was observed in the periodontitis group by comparison to controls, with a trend to decrease after NSPT, suggesting that the oral microbiota tends to become similar to that found in HIV‐1 patients without periodontitis. These results are consistent with those reported earlier about microbial dysbiosis in PLWH with periodontitis [9, 12, 13, 18, 24]. The highly significant improvement in PPD, BOP, and CAL 30 days after NSPT in our panel of periodontitis patients is thought to contribute to reestablishing the homeostasis of the oral microbiota in a controlled HIV‐1 infection, while effective cART may mitigate periodontal risk [27, 28].

The role of the oral microbiota in systemic inflammation has been widely discussed. Enhanced ability to induce pro‐inflammatory cytokines and other inflammation by‐products have been described [24], and an association between oral and gut microbiota, especially in HIV‐1‐infected patients, has been found as they become connected through immune‐driven barrier disruption, translocation of microbes or products, and niche‐specific inflammation that propagates systemic immune activation [13, 28, 29]. In the present study, even if markers of local and systemic inflammation did not show significant differences in saliva or blood between periodontitis and control patients (data not shown), correlations between seven inflammatory markers and seven bacterial taxa particularly associated to periodontitis [21–23, 30–33] were found for saliva in the group of periodontitis patients, which was not the case in controls; interestingly, the profiles after NSPT showed intermediate results. By contrast to saliva, the inflammatory markers of periodontitis patients showed no marked differences in blood plasma, except for TNF‐alpha and partly IL‐4. While the Socransky complexes [34] were originally used to describe bacterial taxa specifically associated with periodontitis, current evidence has demonstrated that several of these taxa are not restricted to periodontitis sites and that many of these species occupy a shared central role across health, gingivitis, and periodontitis [3, 22, 32, 33], suggesting that their presence is not exclusive to periodontitis but rather that they could act as bridge or core organisms for the establishment of dysbiotic microbial communities. Finally, our results show the interplay between the presence of dysbiosis in PLWH with periodontitis, increased abundance of periodontopathogenic bacterial species and oral cytokine‐driven inflammation.

This study presents some limitations. First, a group of controls seronegative for HIV‐1 would have been useful to better understand the weight on the oral microbiota of this chronic infection, even when it is controlled by effective cART. Second, the period of inclusion was considerably delayed by the occurrence of the Covid‐19 pandemic, which complicated the recruitment of participants and the evaluation of data. Third, the small size of the effectives could explain the lack of power of some statistical comparisons, notably concerning the influence of different combinations of antiretroviral drugs; indeed, the benefit of integrase strand transfer inhibitors (ISTIs) on gut microbiota [35] but not on oral microbiota [36] could not be investigated in the present study. Finally, an elevated BOP percentage mean (Table 2), which may denote gingivitis in some individuals [37], was observed in the control group; this finding could relativize the difference in oral health between the two groups of participants. Therefore, as previously shown [38, 39], longer follow‐up periods would have provided additional insight into the evolution of the oral microbiota and periodontal inflammation, in order to confirm whether the observed differences persisted over time. These limitations illustrate the difficulty for selecting the subjects who are voluntary to enter into this kind of comparative trial.

## 5. Conclusions

Our results demonstrate that, in PLWH with controlled immunological status under effective cART, dysbiosis represents the main feature of oral microbial alterations, instead of changes for individual pathogens. Thus, periodontitis patients exhibit enrichment of periodontitis‐related taxa whereas NSPT is effective in improving clinical parameters and reducing microbial diversity towards a healthier profile. Correlations between pathogenic bacteria and pro‐inflammatory cytokines highlight the interplay between microbiota, local immunity, and oral inflammation. In view of the fact that oral microbial imbalance may contribute not only to local disease but also to systemic immune activation in the course of HIV‐1 infection, previous data warrant reinforcing the maintaining of periodontal health as a significant contribution to a comprehensive management of PLWH.

## Author Contributions

Diana Estefania Ramos Peña contributed to collection of data, investigation of patients, management of experiments, analysis of data, and writing of the original manuscript. Oulfa Boussetta‐Charfi contributed to participation in experiments, conception of figures, and data analysis. Angéline Antezack, Philippe Colson, and Virginie Monnet‐Corti were responsible for data analysis and review/editing of the manuscript. Nacera Amroune and Rafael Simone Saia contributed to participation in experiments. Johann Guillemot contributed to participation in experiments and data analysis. Bruno Pozzetto was responsible for data analysis and writing of the manuscript. Sylvie Pillet and Bernard La Scola were responsible for conception of the study, data analysis, and review/editing of the manuscript. Thomas Bourlet and Ana Carolina Fragoso Motta were responsible for conception of the study, project administration, and review/editing of the manuscript.

## Funding

This study was supported by grants from the São Paulo Research Foundation (FAPESP), Brazil (Grants 2018/23031‐2, 2020/07862‐1, and 2022/08308‐3), the National Council for Scientific and Technological Development (CNPq), Brazil (Grant 404092/2016‐0), the Coordination for the Improvement of Higher Education Personnel (Coordenação de Aperfeiçoamento de Pessoal de Nivel Superior, CAPES), Brazil (Finance Code 001), and the University of São Paulo and French Committee for the Evaluation of University‐Level Cooperation with Brazil Program (Grant USP/COFECUB/2017), Brazil–France.

## Conflicts of Interest

Bernard La Scola and Philippe Colson are scientific advisors of BioSellal and Triber companies. The other authors declare no conflicts of interest.

## Data Availability

Data are available upon request due to privacy/ethical restrictions.
